# LRP1 is required for novobiocin-mediated fibronectin turnover

**DOI:** 10.1038/s41598-018-29531-2

**Published:** 2018-07-30

**Authors:** Natasha Marie-Eraine Boel, Morgan Campbell Hunter, Adrienne Lesley Edkins

**Affiliations:** Biomedical Biotechnology Research Unit, Department of Biochemistry and Microbiology, Rhodes University, Grahamstown, 6140 South Africa

**Keywords:** Chaperones, Proteolysis

## Abstract

Fibronectin (FN) plays a major role in the stability and organization of the extracellular matrix (ECM). We have previously demonstrated that FN interacts directly with Hsp90, as well as showing that the Hsp90 inhibitor novobiocin results in FN turnover via a receptor mediated process. However, the receptor involved has not been previously identified. LRP1 is a ubiquitous receptor responsible for the internalisation of numerous ligands that binds both Hsp90 and FN, and therefore we investigated whether LRP1 was involved in novobiocin-mediated FN turnover. FN, LRP1 and Hsp90 could be isolated in a common complex, and inhibition of Hsp90 by novobiocin increased the colocalisation of FN and LRP1. Novobiocin induced an increase (at low concentrations) followed by a loss of FN that was primarily derived from extracellular matrix-associated FN and led to a concomitant increase in intracellular FN. The effect of novobiocin was specific to LRP1-expressing cells and could be recapitulated by an LRP1 blocking antibody and the allosteric C-terminal Hsp90 inhibitor SM253, but not the N-terminal inhibitor geldanamycin. Together these data suggest that LRP1 is required for FN turnover in response to Hsp90 inhibition by novobiocin, which may have unintended physiological consequences in contexts where C-terminal Hsp90 inhibition is to be used therapeutically.

## Introduction

The extracellular matrix (ECM) is constantly remodelled to carry out functions involved in structural support and cell signalling^1^. ECM homeostasis is maintained through a tightly controlled interplay between synthesis, deposition and degradation of matrix components, the deregulation of which has been linked to various pathological diseases^2,3^. Among the ECM proteins, fibronectin (FN) plays important roles in cell adhesion, migration, wound healing and oncogenic transformation^4,5^. FN is produced intracellularly as a soluble protein which is polymerized in an integrin-dependent mechanism into insoluble extracellular fibrillar structures that form the bulk of the ECM^5–7^.

Recently, Heat Shock Protein 90 kDa (Hsp90) was shown to regulate FN matrix stability^8^. Hsp90 is a ubiquitously expressed molecular chaperone which facilitates protein homeostasis in cells^9,10^. Hsp90 is known to be upregulated in cancers and is required for the activation and maturation of oncogenic proteins^11–14^. Hsp90 in the extracellular space mediates cell migration and contributes to metastasis^12,15–18^. Hsp90 and FN interacted directly *in vitro* and in breast cancer cell lines, and Hsp90 depletion by RNA interference or inhibition with the C-terminal inhibitor novobiocin (NOV) induced FN internalisation by a receptor-mediated pathway^8^. However, the receptor mediating this turnover was not identified. LRP1 is a type I transmembrane receptor of the low density lipoprotein (LDL) receptor family^19^. LRP1 is known to be a scavenger receptor as it mediates the internalisation of a diverse range of ligands including proteinases, ECM proteins, bacterial toxins and viruses^20–22^. Studies by Salicioni and colleagues have shown that FN accumulates in the extracellular space in LRP1-deficient CHO/MEF cells, and that LRP1 may serve as a catabolic receptor for FN^23^. In addition to this role, LRP1 interacts with extracellular ligands to promote cell signalling to modulate cellular processes such as migration^24^. Extracellular Hsp90 (eHsp90) is one such ligand of LRP1^25^. Studies have demonstrated that eHsp90 utilizes a unique transmembrane signalling mechanism to promote cell motility and wound healing by binding to LRP1 and activating Akt kinases^26,27^. Several groups have also reported roles for eHsp90 binding LRP1 in cell migration by activating various downstream signalling pathways including ERK, MMP2/9, NFkB^26,28–34^.

The dynamics of FN matrix assembly and degradation play a large role in cell migration and invasion contributing to the metastatic potential of cancer cells. Thus, considering our previous study established a role for Hsp90 in FN matrix dynamics, and that both FN and Hsp90 interact with LRP1, we hypothesised that the LRP1 receptor was involved in the turnover of FN in response to Hsp90 inhibition by NOV. Herein, we report that a trimeric cell surface complex containing Hsp90, LRP1 and FN exists, and that LRP1 is required for the turnover of FN upon Hsp90 inhibition with NOV. Whether Hsp90 acts to chaperone FN to LRP1 in this space or rather serves a cytokine-like role is still unclear.

## Results

### Loss of extracellular FN in response to NOV is rescued by Hsp90β

**Figure 1:**
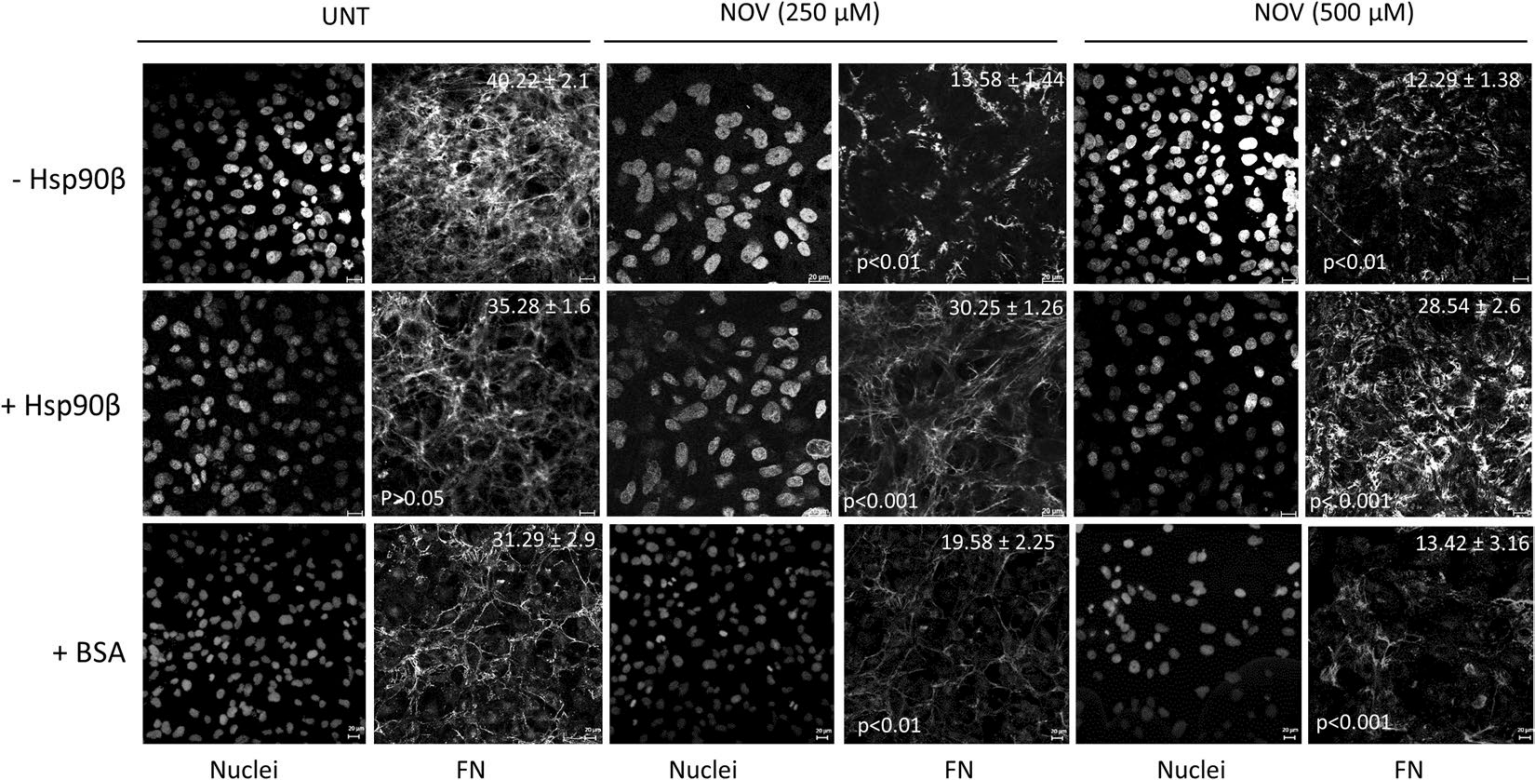
Treatment of Hs578T cells with NOV results in a loss of the FN matrix which is partially rescued by exogenous Hsp90β. Hs578T cells either remained untreated or were pre-treated with novobiocin (NOV; 250 or 500 μM) for 1 hour followed by addition of media with exogenous endotoxin-free Hsp90β (+Hsp90β; 100 ng/ml) or without (−Hsp90β) or with exogenous endotoxin-free BSA (+BSA; 100 ng/ml) overnight. Fixed cells were stained using mouse anti-human FN followed by donkey anti-mouse DyLight® 488 secondary antibody. Nuclei were stained with Hoechst 33342 (1 μg/ml). Images were captured using a Zeiss LSM 510 Meta laser scanning confocal microscope. Images were analyzed using Zen software, blue edition (Zeiss, Germany). Scale bars are equivalent to 20 μm. Values (in white) at the top of each frame represent the mean grey values per nuclei ± SD (n = 3) for each treatment. Mean grey values were compared using a one-way ANOVA with a Tukey Post Test. The mean grey value of NOV treatments (NOV; 250 or 500 μM) were compared to untreated cells for both negative controls (−Hsp90β and +BSA). The mean grey value of exogenous Hsp90β (+Hsp90β) treated cells were compared to the equivalent treatment with BSA (+BSA). Statistical p-values are shown in the bottom left of each frame. The data shown are representative of triplicate images collected from duplicate independent experiments.

We first tested the effect of Hsp90 inhibition with NOV on the extracellular FN matrix. Hs578T breast cancer cells (which endogenously express high levels of FN matrix) were treated with or without increasing concentrations of NOV and the resulting FN phenotype observed. The ability of extracellular Hsp90 to rescue the observed phenotype was tested by addition of exogenous endotoxin-free Hsp90β (Fig. 1). Treatment with BSA, a non-specific protein that does not bind either LRP1 or NOV, served as a control for the addition of Hsp90β. The average FN fluorescence intensity per cell number (measured by the number of nuclei) in multiple images was quantified using ImageJ in order to compare the FN staining between samples. Hs578T cells showed a statistically significant and dose dependent decrease in the extracellular FN matrix upon treatment with increasing concentrations of NOV compared to the untreated (UNT) cells in both the presence of BSA (Fig. 1, bottom panel) and absence of Hsp90β (Fig. 1, top panel). There was a significant (p < 0.001) recovery of the extracellular FN matrix upon addition of exogenous Hsp90β to NOV treated cells (Fig. 1, middle panel). Treatment of Hs578T cells with Hsp90β alone showed no significant increase in the extracellular FN matrix, although better defined FN matrix fibrils were observed.

### NOV increased colocalisation of FN and LRP1

**Figure 2:**
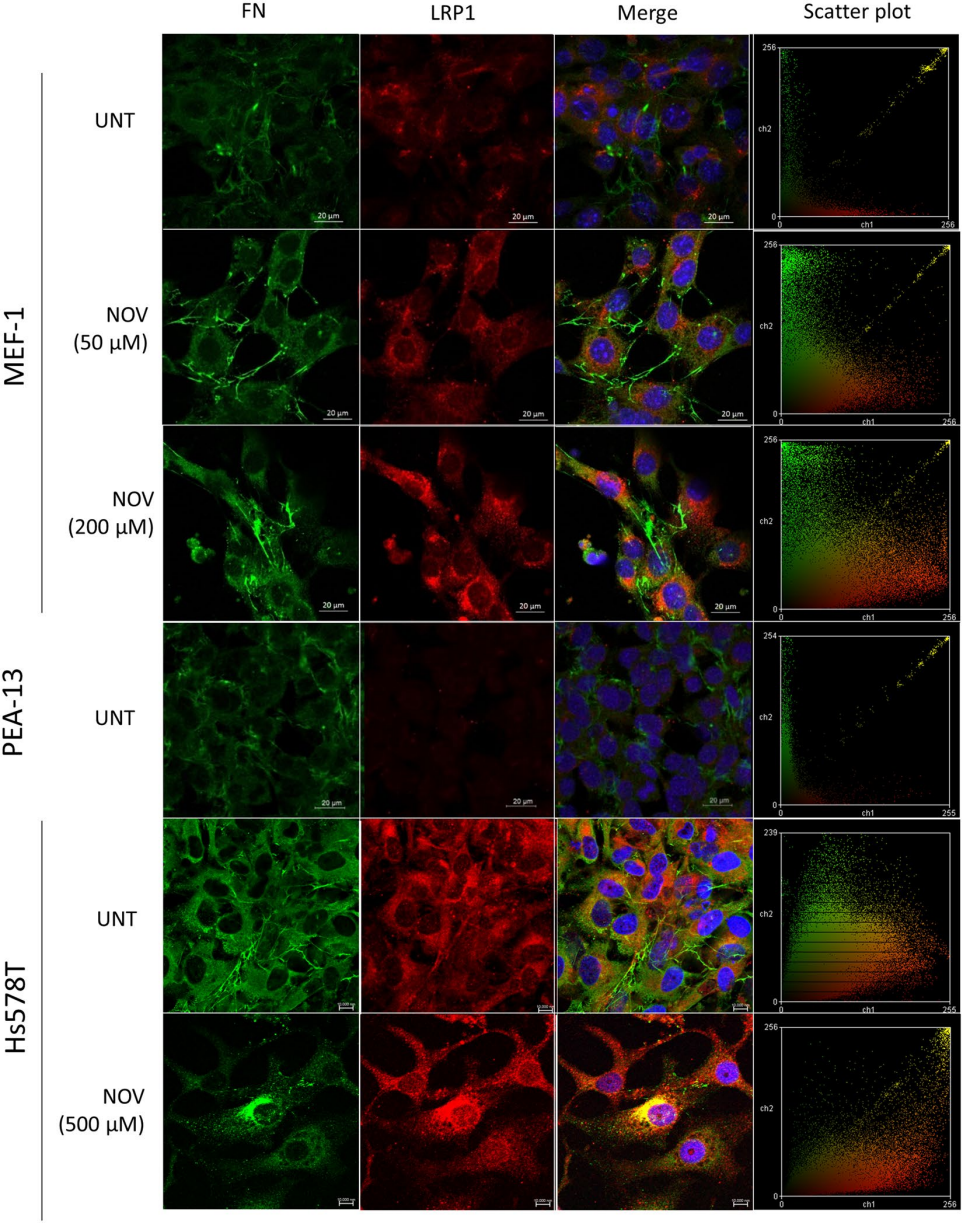
NOV treatment increased FN and LRP1 colocalisation in MEF-1 and Hs578T cells. Cells were treated with increasing concentrations of novobiocin (NOV) for 16 hours. Cells were fixed and incubated with mouse anti-FN (green) and rabbit anti-LRP1 (red) primary antibodies followed by donkey anti-mouse Alexa Fluor-488 and donkey anti-rabbit Alexa Fluor-546 respectively. Nuclei were stained with (1 μg/ml) Hoechst-33342 (blue). Images were captured using the 63x objective on the Zeiss LSM 780 Meta laser scanning confocal microscope and analyzed using Zen Blue software (Zeiss, Germany). Scatter plots were generated using Intensity Correlation Analysis plugin in ImageJ. Data are representative of images obtained from triplicate independent experiments with similar results. Scale bars represent 20 μm.

**Figure 3:**
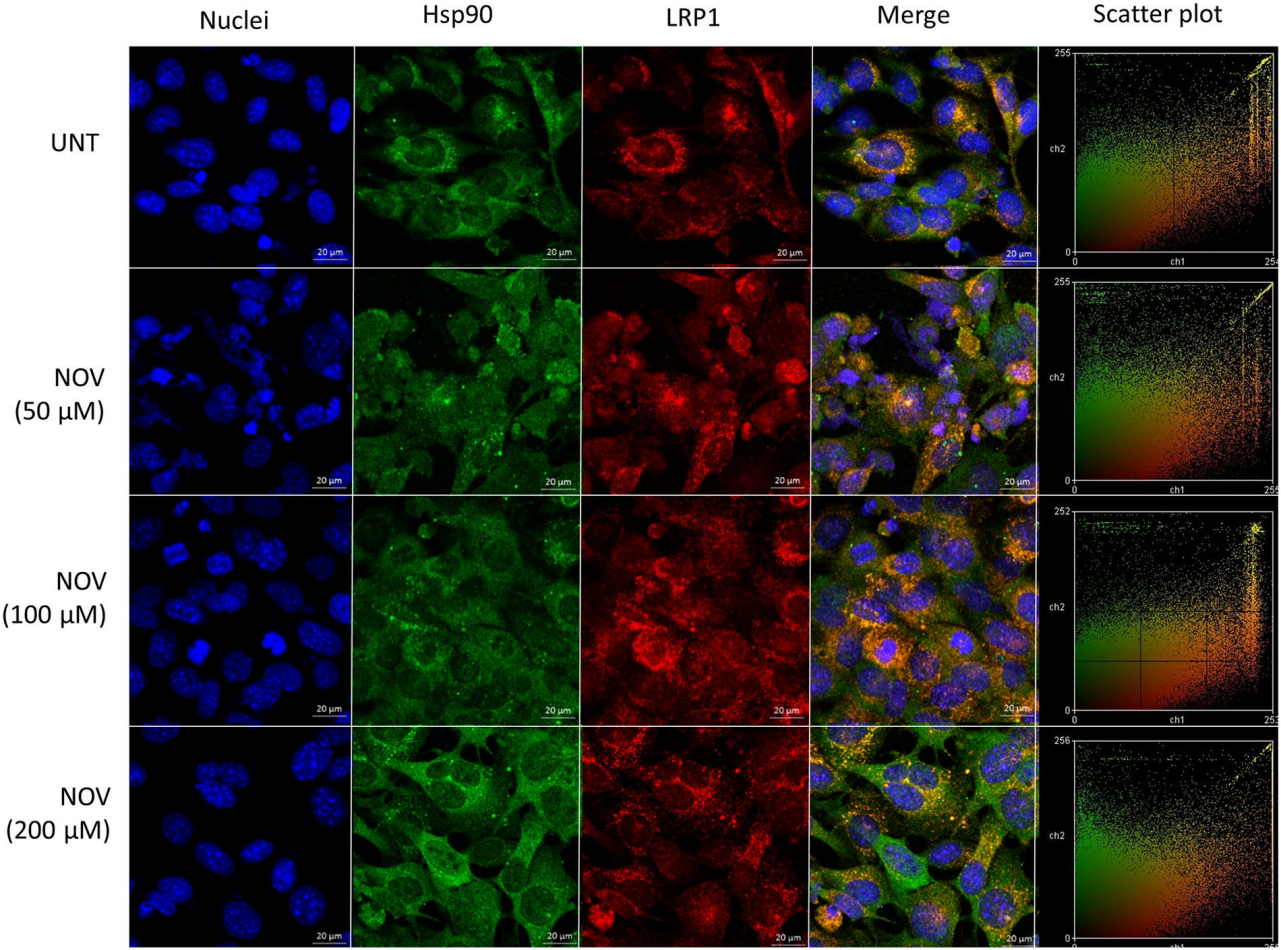
LRP1 and Hsp90 colocalise upon NOV treatment in MEF-1 cells. MEF-1 cells were treated with increasing concentrations of novobiocin (NOV) for 16 hours. Cells were fixed and incubated with goat anti-human Hsp90α/β (green) and rabbit anti-human LRP1 (red) followed by donkey anti-goat Alexa Fluor-660 and donkey anti-rabbit Alexa Fluor-546. Nuclei were stained with (1 μg/ml) Hoechst-33342 (blue). Images were captured using the 63x objective on the Zeiss LSM 780 Meta laser scanning confocal microscope and analyzed using Zen Blue software (Zeiss, Germany). Scatter plots were generated using Intensity Correlation Analysis plugin in ImageJ. Data are representative of images obtained from triplicate independent experiments with similar results. Scale bars represent 20 μm.

**Figure 4:**
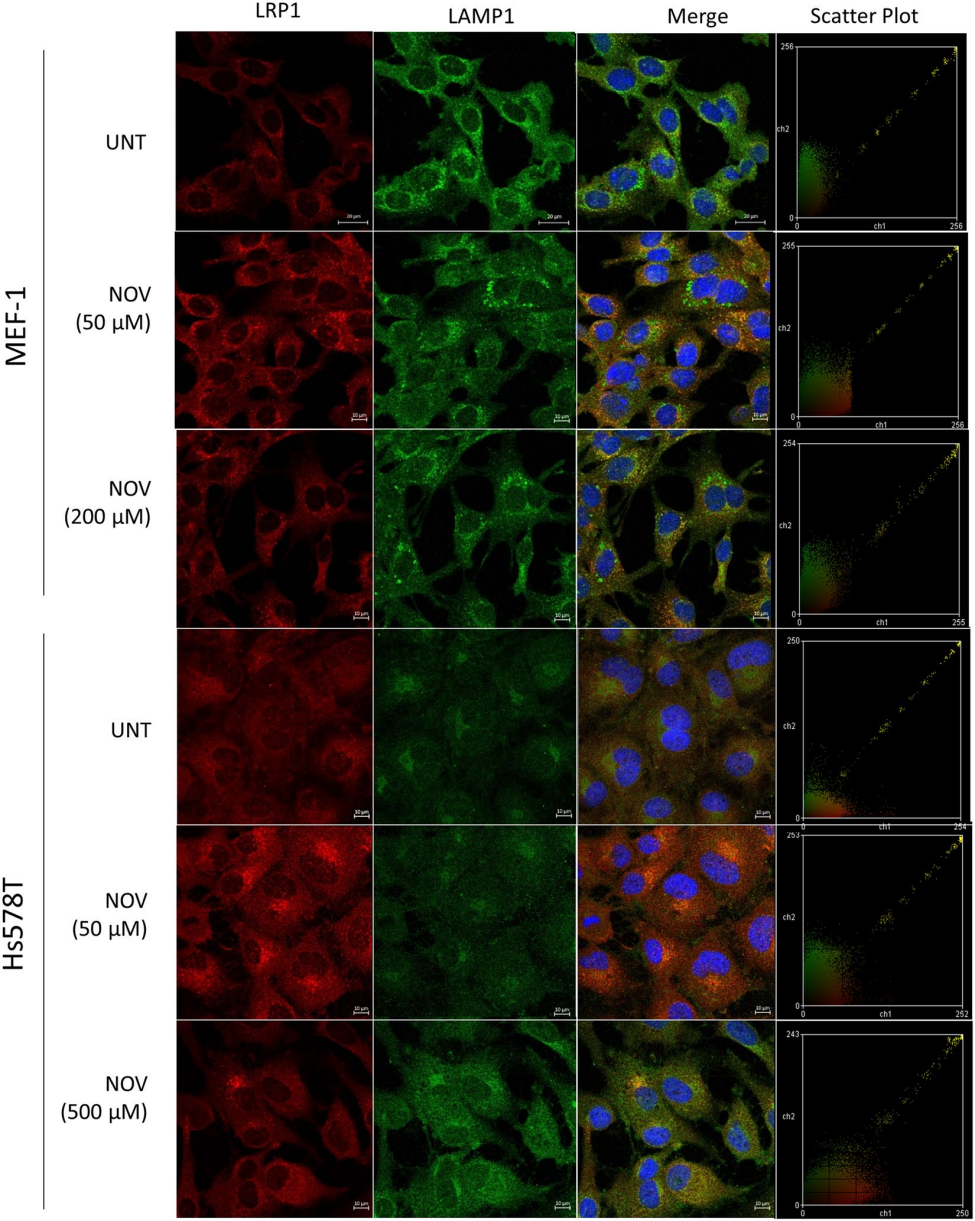
NOV treatment increased LRP1 and LAMP1 colocalisation in MEF-1 and Hs578T cells. Cells were treated with increasing concentrations of novobiocin (NOV) for 16 hours. Cells were fixed and incubated with rabbit anti-LRP1 (red) primary antibody followed by donkey anti-rabbit Alexa-fluor-488 and mouse anti-LAMP1 Alexa Fluor 630 (green). Nuclei were stained with (1 μg/ml) Hoechst-33342 (blue). Images were captured using the 63x objective on the Zeiss LSM 780 Meta laser scanning confocal microscope and analyzed using Zen Blue software (Zeiss, Germany). Scatter plots were generated using Intensity Correlation Analysis plugin in ImageJ. Data are representative of images obtained from triplicate independent experiments with similar results. Scale bars represent 20 μm.

As both Hsp90 and FN interact with LRP1, and the Hs578T cell line expresses LRP1, we hypothesised that this receptor may be involved in Hsp90-mediated FN turnover. We therefore investigated whether LRP1 colocalised with FN (Fig. 2) and/or Hsp90 (Fig. 3) and/or the lysosomal marker LAMP-1 (Fig. 4) in NOV-treated cells by confocal microscopy. In addition to the Hs578T cell line, we also used an isogenic mouse embryonic fibroblast (MEF) model system of differential LRP1 expression^35,36^. The MEF1 cell line is derived from wild type mice, while the PEA13 cell line are isogenic lines from LRP1 knockout mice. Colour Scatter Plots generated shows plots of green intensities vs. red intensities with yellow indicative of overlapping green and red pixels. In both MEF-1 and Hs578T cells, increasing NOV treatment appeared to increase the colocalisation of FN and LRP1 in a dose dependent manner compared to untreated cells (Fig. 2). A diffuse staining pattern of Hsp90 was observed in all NOV treatments (Fig. 3) and there was colocalisation between Hsp90 and LRP1 in both untreated and NOV treated MEF-1 cells as observed by yellow pixels in merged images. Furthermore, we demonstrate that LRP1 is internalised in both Hs578T and MEF-1 cells (Fig. 4) by co-staining with the endocytic marker, LAMP-1. In addition, in response to NOV treatment we observed an increase in the intensity of intracellular LRP1 staining and colocalisation with LAMP1 in both cell lines. Taken together, these data suggest that NOV treatment results in LRP1 endocytosis increasing colocalisation of LRP1 and FN and LRP1 and Hsp90 in lysosomes.

### LRP1, Hsp90 and FN can be isolated in a common complex

**Figure 5:**
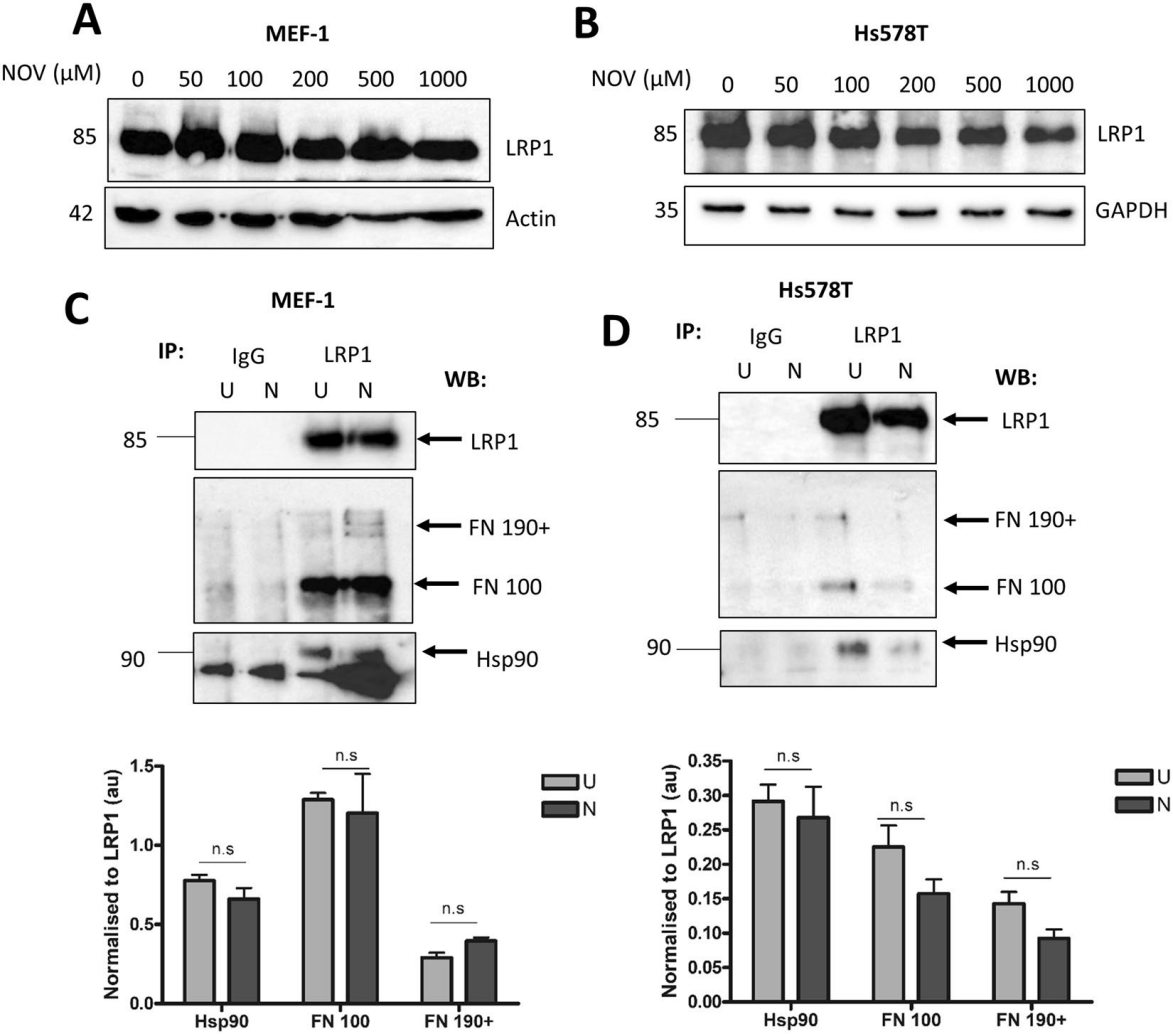
Hsp90, LRP1 and FN occur in a common complex in MEF-1 and Hs578T cells. (**A**) MEF-1 and (**B**) Hs578T lysates were treated with NOV and probed for LRP1 using rabbit anti-LRP1 antibodies. Actin or GAPDH were used as loading controls respectively. (**C**) MEF-1 and (**D**) Hs578T cells were left untreated (U) or treated with 50 μM NOV (N) for 16 hours. Extracellular proteins were crosslinked with the cell-impermeable crosslinker, DTSSP, and LRP1-containing complexes were isolated by immunoprecipitation (IP) with either LRP1 or isotype control IgG antibodies. Elution fractions were resolved on a 10% SDS gel and probed for FN, LRP1 and Hsp90 using rabbit anti-FN, rabbit anti-LRP1 and mouse anti-Hsp90 primary antibodies respectively. Densitometry of expression levels of FN and Hsp90 were determined relative to the amount of LRP1 in each immunoprecipitation and are presented as bar graphs. Statistical analyses was performed using a one-way ANOVA with Bonferroni post-test. Data shown are representative of duplicate experiments with similar results.

LRP1 is known to bind Hsp90^25^ and studies have demonstrated LRP1 to be a catabolic receptor for the uptake of FN^23^. The association of Hsp90 with FN has previously been described by our group^8^. We therefore tested for a putative complex between Hsp90, LRP1 and FN (Fig. 5). We first showed that both MEF-1 and Hs578T cells express LRP1 and that levels of LRP1, unlike FN, are not affected by NOV treatment (Fig. 5A,B). NOV-treated and untreated MEF-1 and Hs578T cells were incubated with the cell-impermeable DTSSP crosslinker to covalently crosslink cell surface proteins and crosslinked LRP1 containing complexes isolated by immunoprecipitation (Fig. 5). LRP1 co-immunoprecipitation showed that Hsp90 and FN occur in a common complex with LRP1 on the surface of MEF-1 and Hs578T cells (Fig. 5C,D) in agreement with the colocalisation of FN, LRP1 and Hsp90 observed in the confocal analysis (Figs 2 and 3). In the immunoblot for FN in MEF-1 cells (Fig. 5C), immunoblotting for LRP1 showed similar levels of LRP1 (85 kDa) confirming that equal amounts of LRP1 were isolated in both fractions. The absence of proteins detected in the IgG IP elution fractions confirmed there were no non-specific binding of proteins. Upon overexposure, similar levels of Hsp90 were detected in both the NOV (N) and untreated (U) fractions as quantified in bar graphs below (Fig. 5C). The bands below Hsp90 represent non-specific binding. Treatment of Hs578T cells revealed similar results (Fig. 5D). Untreated and NOV treated lysates yielded similar levels of FN and Hsp90 immunoprecipitated with LRP1 (Fig. 5D). These data support the presence of a common complex containing Hsp90, LRP1 and FN in both Hs578T and MEF-1 cells that exists in both the absence and presence of NOV.

### LRP1 blocking antibody recapitulated the effect of NOV on the FN matrix and was rescued by Hsp90β

**Figure 6:**
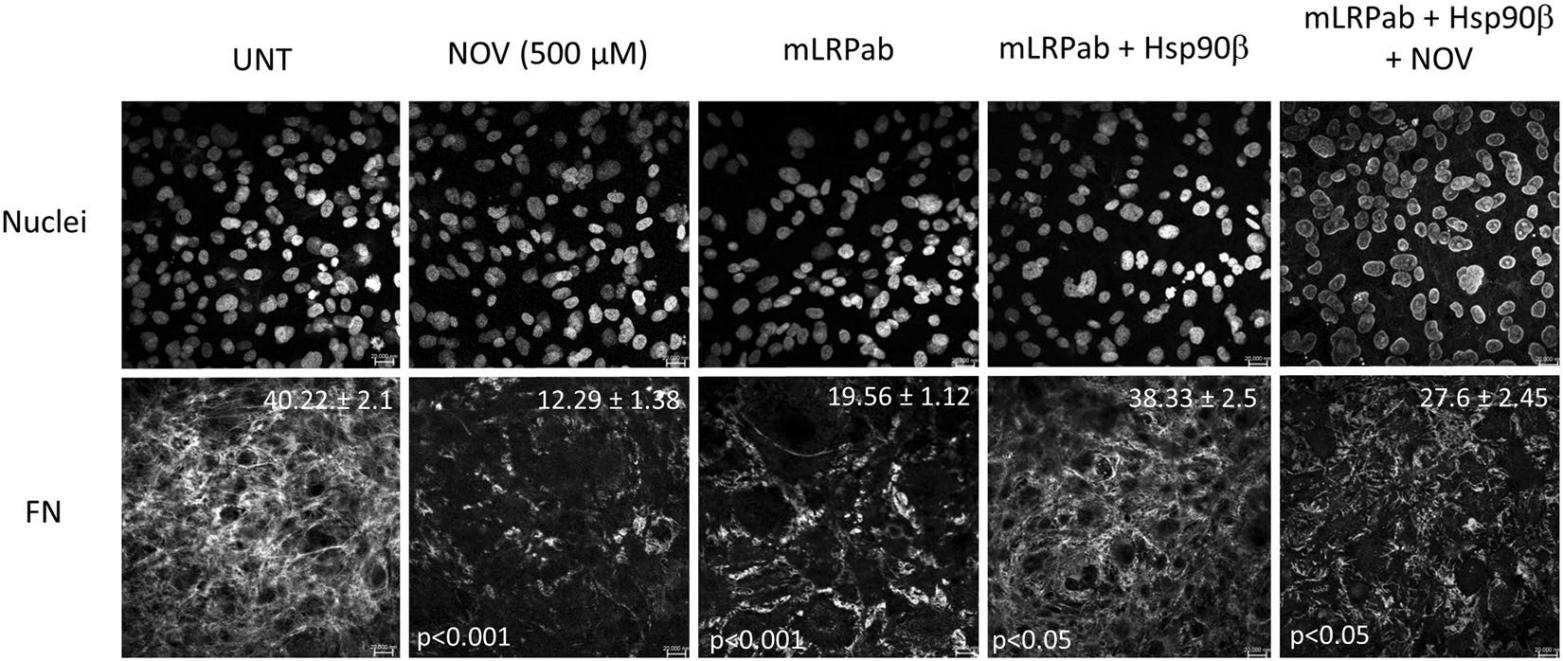
LRP1 blocking antibody induced loss of FN matrix which could be rescued by soluble Hsp90β. Hs578T cells were treated with novobiocin (NOV; 500 μM) for 1 hour at 37 °C followed by treatment with a blocking LRP1 antibody (mLRPab; 2 μg/ml) for 10 minutes before addition of exogenous Hsp90β (100 ng/ml) overnight. Fixed cells were stained using rabbit anti-human FN followed by donkey anti-rabbit DyLight® 488 fluorescent secondary antibodies. Nuclei were stained with Hoechst 33342 (1 μg/ml). Images were captured using a Zeiss LSM 510 Meta laser scanning confocal microscope and analyzed using Zen software, blue edition (Zeiss, Germany). Scale bars are equivalent to 20 μm. Values (in white) at the top of each frame represent the mean grey values per nuclei ± SD (n = 3) for each treatment. Mean grey values were compared using a one-way ANOVA with a Tukey Post Test comparing all values. The mean grey value of NOV and LRP1 blocking antibody (mLRPab) treatments were compared to untreated cells. The mean grey value of LRP1 blocking antibody and exogenous Hsp90β treated (mLRPab + Hsp90β) cells were compared to the equivalent treatment without exogenous Hsp90β (mLRPab). The mean grey value of a combination of all three treatments (NOV + mLRPab + Hsp90β) was compared to untreated cells. Statistical p-values are shown in the bottom left of each frame. The data shown are representative of triplicate images collected from duplicate independent experiments.

Having shown that FN, LRP1 and Hsp90 can be isolated in a common complex, the effect of a commercial monoclonal LRP1 blocking antibody [8G1] on the FN matrix was compared to NOV treatment (Fig. 6). This antibody is reported to bind the 515 kDa alpha chain of LRP1 and prevent ligand binding^20,37^. Treatment of Hs578T cells with the LRP1 blocking antibody (Fig. 6; mLRPab) showed a significant (p < 0.001) loss of extracellular FN in comparison to the untreated control (UNT) and was similar to NOV treatment (Fig. 6). The addition of exogenous Hsp90β was able to significantly overcome the observed FN phenotype in mLRPab treated Hs578T cells (p < 0.05) and restored the extracellular FN matrix phenotype of mLRPab treated Hs578T cells similar to that of the untreated control (Fig. 6, mLRPab + Hsp90). Treatment with a combination of NOV, mLRPab and exogenous Hsp90β resulted in a loss of extracellular FN similar to that of NOV and mLRPab treated Hs578T cells, which support the fact that Hsp90β was responsible for restoring the extracellular FN matrix phenotype due to the mLRPab (Fig. 6).

### Loss of extracellular FN in response to NOV is dependent on the presence of LRP1

**Figure 7:**
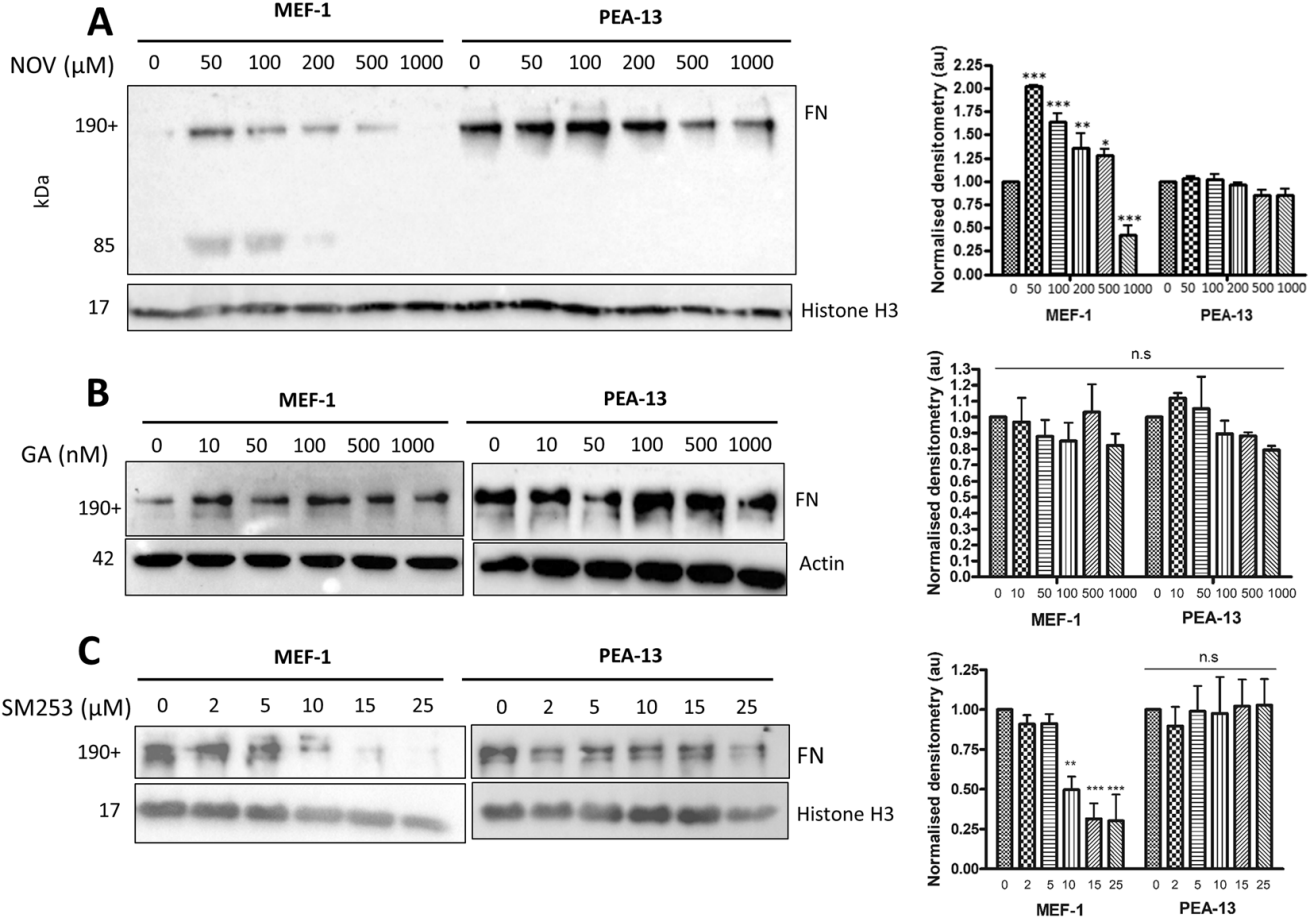
Loss of FN is specific to C-terminal Hsp90 inhibition in LRP1-expressing cells. (**A**) Adherent MEF-1 and PEA-13 were treated with increasing concentrations of novobiocin (NOV), (**B**) geldanamycin (GA) or (**C**) SM253 for 16 hours at 37 °C. Cells were lysed and equal amounts of total protein (50 μg) were probed for levels of FN using rabbit anti-FN primary antibodies. Histone H3 and actin were used as a loading controls. The densitometry represented alongside was determined using ImageJ. Statistical significance was determined using a two-way ANOVA in GraphPad Prism 4 (*p < 0.05, **p < 0.01, ***p < 0.001). Error bars indicate ± SD (n = 3).

**Figure 8:**
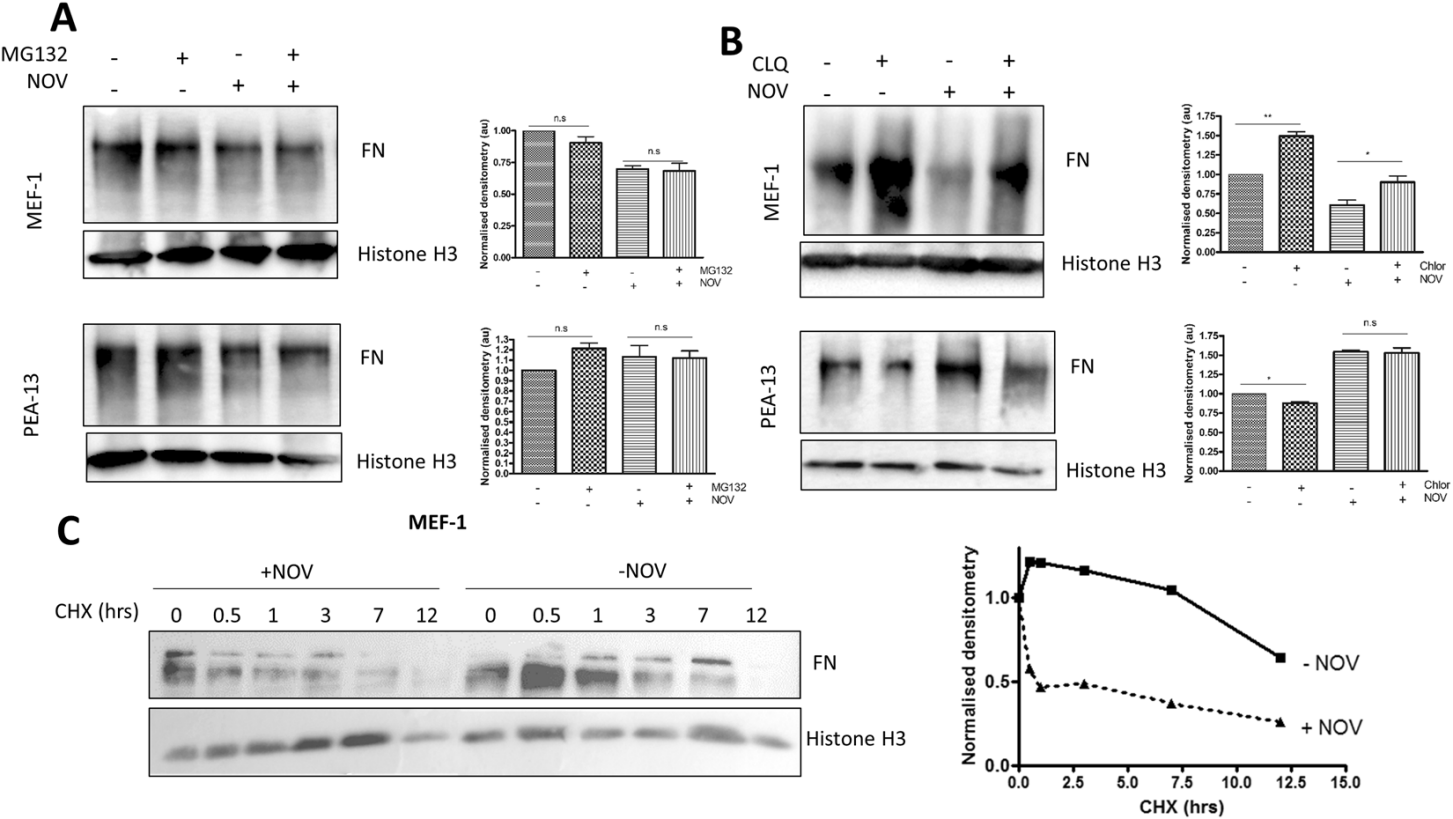
Effect of Hsp90 inhibition on the degradation of FN in MEF-1 and PEA-13 cells. MEF-1 and PEA-13 cells were treated (**A**) with (+) or without (−) MG132 (5 μM) and (**B**) with (+) or (−) without chloroquine (CLQ) (100 μM) in the presence or absence of 200 μM NOV for 16 hours before lysing and probing for levels of FN. Statistical significance was determined by an unpaired two-tailed Student’s t-test in GraphPad and is shown in bar graphs alongside. (**C**) MEF-1 cells were treated with 100 μg/ml cycloheximide (CHX) in the presence or absence of novobiocin (NOV 200 μM) and lysates prepared at the indicated time points. Equal amounts of total protein were probed for FN on a western blot with rabbit anti-FN antibody. Histone H3 was used as a loading control. The associated densitometry was determined using ImageJ software and is presented alongside. Data are representative of triplicate independent experiments.

MEF-1 and PEA-13 cells were treated with increasing doses of NOV (0–1000 µM) and samples were processed for western analysis (Fig. 7A). Immunoblotting for FN revealed that MEF-1 cells had significantly increased total FN levels upon NOV treatment at 50 µM, followed by a dose-dependent loss of FN at higher NOV concentrations (Fig. 7A). Comparatively, LRP1-deficient PEA-13 cells showed no significant changes in total FN levels at equivalent concentrations. Interestingly, in MEF-1 cells, we also detected lower molecular weight FN bands (85 kDa) at low NOV concentrations (Fig. 7A). We suspect these may represent either proteolytic or assembly fragments of FN which importantly were not observed in the PEA-13 cells (Fig. 7A). These results suggest that there is a dose-dependent loss of FN in LRP1-expressing (i.e. MEF-1) cells, while in LRP1-deficient cells total FN levels do not change. We further show that this change in FN is specific to C-terminal inhibition of Hsp90 by demonstrating that Hsp90 inhibition by geldanamycin (GA), a known N-terminal inhibitor (Fig. 7B) does not produce the same response as NOV. Unlike NOV, GA is also known to bind Grp94, the ER Hsp90 paralogue, so these data also verify that the effects observed are not due to Grp94 inhibition. We also tested another C-terminal Hsp90 inhibitor, SM253 (kindly provided by Shelli McAlpine, UNSW, Australia), which blocks the Hsp90 C-terminus allosterically through interaction with the M domain of Hsp90^38^. We demonstrate that SM253 produces a significant dose-dependent loss in FN, similar to that observed for NOV but only in MEF-1 cells and not in the PEA13 cell line (Fig. 7C). The changes in total FN observed in the MEF-1 cells (Fig. 7A) could be due to changes in rates of FN synthesis or degradation. To confirm if NOV resulted in FN degradation, we tested the effect of blocking of proteasomal and lysosomal degradation pathways on levels of FN in these cell lines. Cells were treated with the proteosomal inhibitor, MG132, in the presence and absence of NOV for 16 hours before being lysed and analysed by western analysis (Fig. 8A). In both MEF-1 and PEA-13 cells, addition of MG132 in the presence of NOV caused no significant accumulation of FN relative to NOV or MG132 treatments alone. On the other hand, treatment of MEF-1 cells with the lysosomal inhibitor, chloroquine (+CLQ) (Fig. 8B) significantly increased levels of FN compared to control cells (−CLQ) in both untreated and NOV treated cells. There was no significant increase in FN levels in response to lysosomal inhibition in PEA13 cells. ECM proteins are known to be internalised by receptor mediated endocytosis and degraded in lysosomes^39^. Evidence of FN turnover through its endocytosis and lysosomal degradation have been demonstrated^23^, whilst protease inhibitors were shown to be unable to inhibit FN turnover^40^. Supporting studies have also demonstrated the proteasomal inhibitor to be ineffective in preventing degradation of FN^41^. Given that the main proteolytic pathway for intracellular proteins is the proteasome, while extracellular proteins are predominantly processed by the lysosome^42^, taken together these data could suggest that the observed loss of FN in NOV treated MEF-1 cells (Fig. 7A) is due to internalization and degradation of extracellular FN in the lysosome. These data are consistent with the colocalisation of LRP1 and LAMP1 in NOV treated cells. In order to assess the effect of Hsp90 inhibition on FN protein stability, we performed a time course measurement of FN levels in MEF-1 cells when translation was blocked with cycloheximide (CHX)^43^. MEF-1 cells were treated with CHX in the presence (+NOV) and absence (−NOV) of NOV for the indicated time periods (Fig. 8C). When translation of FN was blocked, a loss in total FN protein levels occurred earlier in NOV treated cells (+NOV) over 12 hours compared to the untreated control (-NOV). When compared with the control (−NOV) where FN levels appear stable with a noticeable loss observed only after 12 hours with CHX alone, levels of FN in NOV and CHX treated lysates were reduced after as little as 0.5 hrs and continued to reduce over the time course of the experiment (Fig. 8C). Additionally, mRNA levels of FN were not significantly altered during NOV treatments (data not shown). Taken together, these data suggest that the dose dependent decrease in total FN levels of MEF-1 cells above 50 μM NOV (Fig. 8C) was most likely due to enhanced turnover of the protein.

### NOV-induced loss of FN is largely extracellular FN matrix

**Figure 9:**
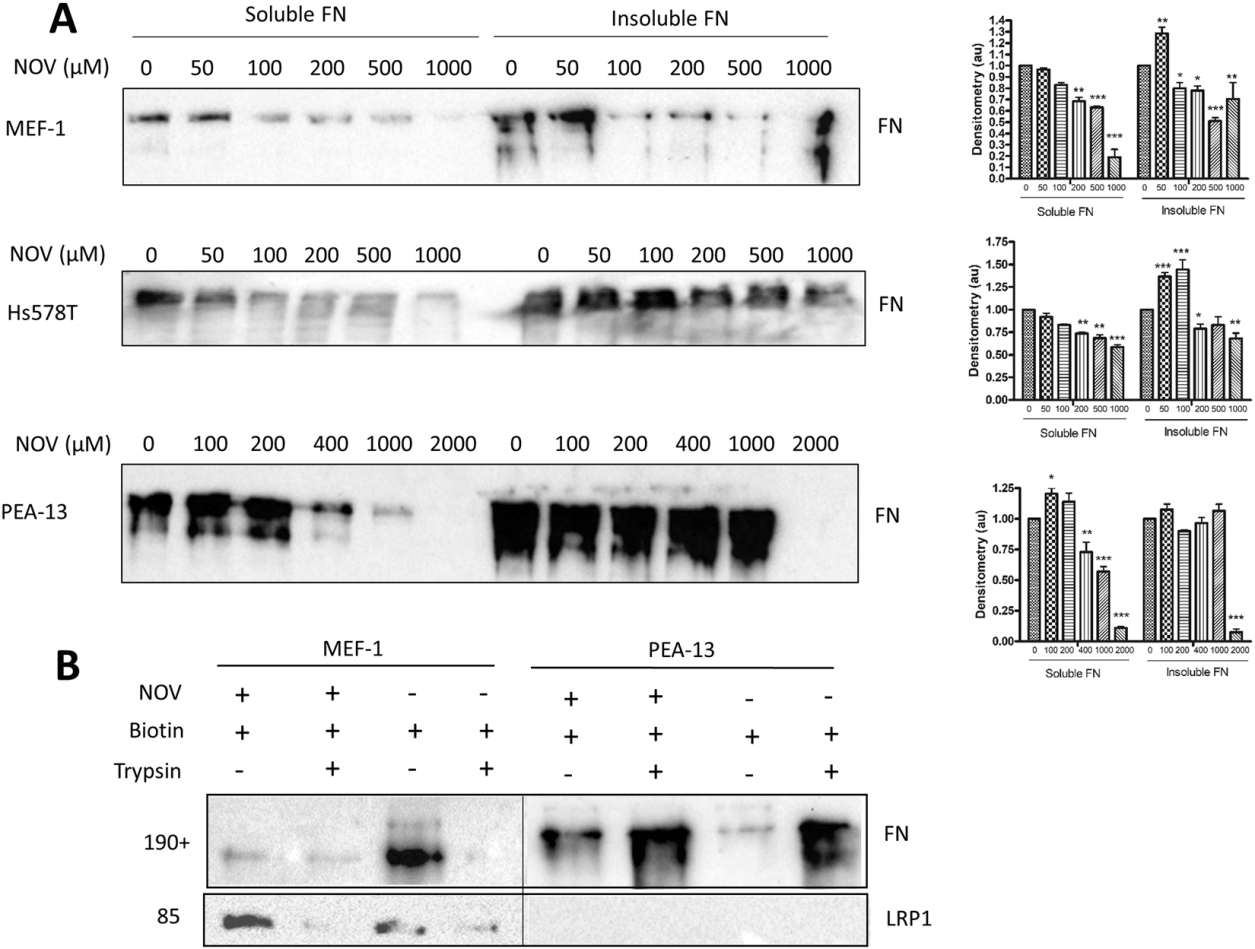
Loss of insoluble extracellular FN in response to NOV in MEF-1 not PEA-13 cells. (**A**) MEF-1, Hs578T and PEA-13 cells were treated with increasing doses of novobiocin (NOV) for 16 hours at 37 °C. Equal numbers of cells were harvested and the soluble and insoluble FN fractions separated using the DOC assay (Brenner *et al*.^44^). FN levels were detected by immunoblotting with rabbit anti-human FN antibody. Densitometry values of the band intensities as determined in ImageJ are indicated in bar graphs representing duplicate experiments presented alongside. Statistical significance was determined compared to the untreated control by a two-way ANOVA with Bonferroni post-test in GraphPad Prism 4 (*p < 0.05, **p < 0.01, ***p < 0.001). (**B**) MEF-1 and PEA-13 cells were treated for 16 hours with novobiocin (NOV 200 μM) or left untreated. Surface biotinylated proteins were purified by streptavidin affinity chromatography and biotinylated fractions from equal numbers of cells and the resultant lysates probed for the presence of FN and LRP1 by western analysis. The data shown are representative of triplicate independent experiments.

In an attempt to identify whether the loss of total FN observed was due primarily to changes in the extracellular insoluble levels of FN, we employed two different assays to isolate this fraction of FN. First, a DOC assay was performed (Fig. 9A) in order to fractionate DOC-soluble (cell-associated) and DOC-insoluble (matrix-associated) FN pools^44^. These fractions were probed for FN by western analysis and revealed increased levels of both insoluble and soluble FN in PEA-13 cells in comparison to MEF-1 cells (Fig. 9A). Upon NOV treatment, the insoluble FN in the LRP1-expressing MEF-1 and Hs578T cells increased at low NOV concentrations (i.e. 50 µM) followed by a decrease to levels below that of the untreated control. The soluble levels of FN in this cell line also decreased in a dose dependent manner. There was no change in the insoluble FN levels of PEA-13 cells suggesting a resistance to extracellular FN loss in response to NOV treatment. Only at the highest concentration (2 000 μM NOV) was the insoluble FN fraction lost. Soluble FN levels were stable up to 200 μM, after which there was a dose dependent decrease (Fig. 9A). These data suggest that NOV treatment induces the loss of insoluble extracellular FN in LRP1-expressing MEF-1 and Hs578T cells, but not in LRP1-deficient PEA-13 cells.

In the second assay, we used a biotin-streptavidin purification to isolate extracellular surface proteins which had been biotinylated (Fig. 9B). MEF-1 and PEA-13 cells were treated with or without NOV (200 µM) before cell surface proteins were labelled by biotin tagging with the cell impermeable EZ-link NHS-biotin^8^. Figure 9B shows the western analysis of the affinity purified fractions with (+) and without (−) NOV and/or trypsin treatment (to demonstrate that extracellular fractions were isolated). The biotinylated MEF-1 fractions revealed reduced levels of FN in (+NOV) samples compared to (−NOV) samples. Equivalent control fractions which had been trypsin treated resulted in an almost complete loss of biotinylated FN. LRP1 levels were also reduced in response to trypsin treatment confirming that surface proteins were being isolated (Fig. 9B). Biotinylated PEA-13 fractions revealed increased surface FN in NOV-treated samples compared to (−NOV). Interestingly, affinity purified FN in PEA-13 cells appeared to be more resistant to trypsin treatment (+trypsin) in comparison to MEF-1 cells and in fact displayed higher levels of FN than the (−trypsin) fractions. The increased resistance of LRP1-deficient cells to trypsin detachment has been noted by others^45^.

**Figure 10:**
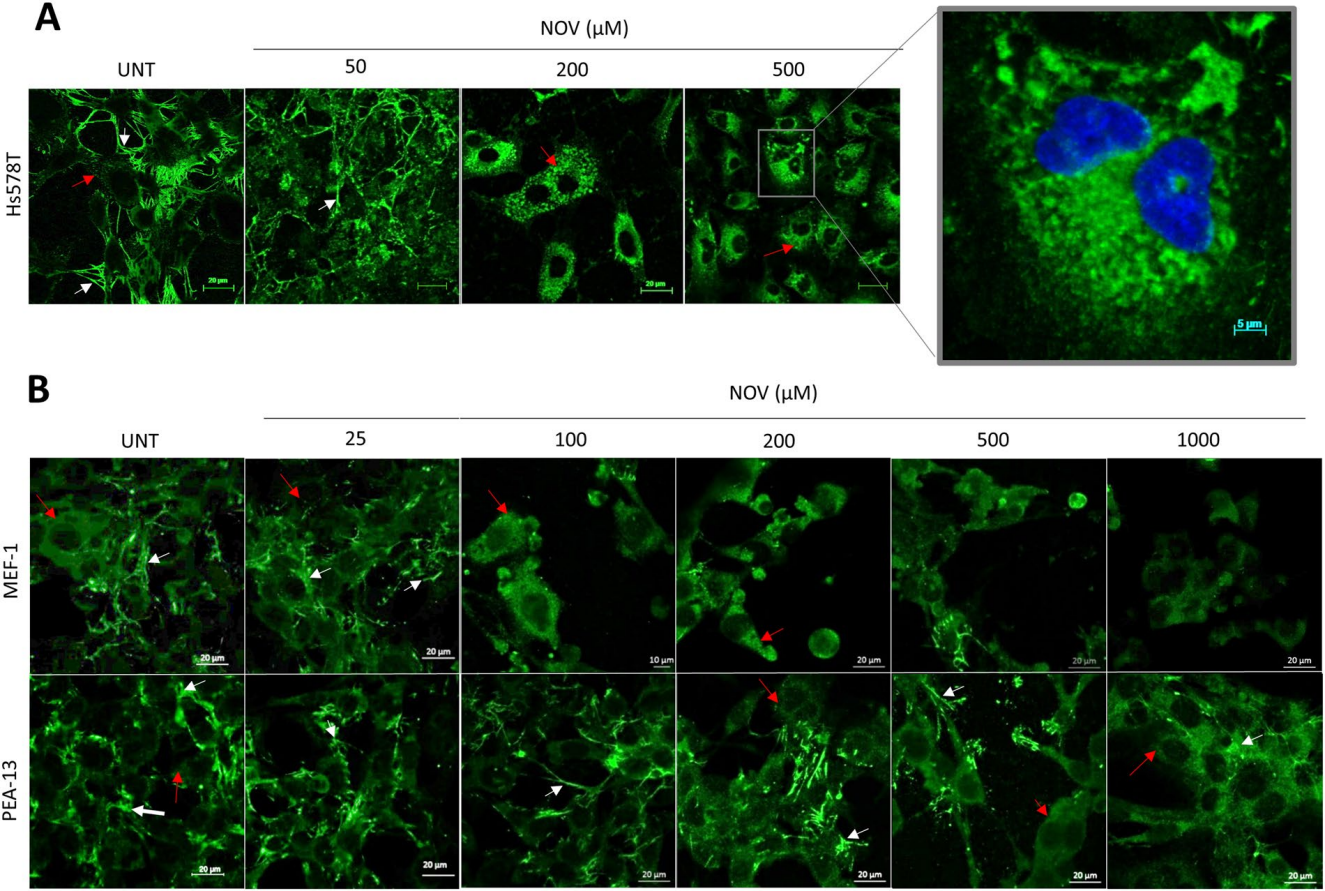
NOV induces internalisation of fibronectin matrix in LRP1-expressing Hs578T and MEF-1 cells but not LRP-deficient PEA13 cells. Cells were allowed to adhere overnight in a glass bottomed 15 well ibidi microdish and were treated with increasing concentrations of NOV in (**A**) Hs578T cells (0–500 μM) and (**B**) MEF-1 and PEA-13 cells (0–1000 μM) for 16 hours at 37 °C. Cells were fixed with ethanol and incubated with mouse primary antibody against FN (ab194395) followed by donkey anti-mouse Alexa Fluor-488. Images were captured using the Zeiss LSM 780 Meta laser scanning confocal microscope with the 63x oil objective and analysed using Zen Blue software. White arrows indicate extracellular FN matrix or FN fibrils and red arrows indicate intracellular FN. Scale bars represent 20 μm. The data shown are representative of results obtained from triplicate experiments in all cases.

The loss of extracellular FN was confirmed by confocal microscopy. Untreated Hs578T (Fig. 10A), MEF-1 and PEA-13 (Fig. 10B) cell lines had a distinct FN fibrillar network in the extracellular space between neighbouring cells (indicated by white arrows). Low concentrations of NOV (i.e. 50 µM) appeared to maintain, and perhaps even enhance, the appearance of an extracellular FN matrix in LRP1 expressing Hs578T (Fig. 10A) and MEF-1 (Fig. 10B) cells. In Hs578T cells (Fig. 10A), upon higher NOV treatments (200 μM and 500 μM), the matrix was almost entirely lost appearing more intracellular (red arrows), with a concomitant appearance of vesicle-like structures within these cells as depicted in the magnified image (Fig. 10A). Treatment with NOV at higher concentrations in MEF-1 cells revealed a substantial loss of FN matrix (white arrows) with a concomitant increase in intracellular FN (red arrows) (Fig. 10B). PEA-13 cells did not lose their extracellular FN matrix as readily as the MEF-1 cells in response to NOV and some extracellular FN is seen to be retained even at the highest NOV concentration (Fig. 10B).

Collectively these data demonstrate that LRP1-deficient PEA-13 cells have high levels of extracellular FN matrix which is largely insoluble and does not readily decrease upon Hsp90 inhibition with NOV, whilst LRP1-expressing MEF-1 cells have comparatively less extracellular FN, which is more readily internalised upon Hsp90 inhibition.

## Discussion

The ECM is highly dynamic and is constantly being remodelled to accommodate both physiological and pathological activities^3,46^. Tissue homeostasis requires a balance between ECM synthesis and degradation. Any stimulus which perturbs homeostasis may result in progression of various disease states including fibrosis, cancer and other developmental abnormalities^47,48^. We previously identified FN as a client of Hsp90 as it directly binds to Hsp90 and is dependent on Hsp90 for stability and conformational regulation^8^. In Hs578T cells, inhibition of Hsp90 with NOV led to a destabilisation of the FN matrix resulting in FN internalisation. The addition of exogenous Hsp90β and/or endocytosis inhibitors was able to rescue the effect of NOV on FN (Fig. 1)^8^. We and other authors have previously reported that levels of extracellular Hsp90 in breast, colon and brain cancer cell lines range from approximately 5–20 ng/ml per 10^6^ cells^27,49^. Therefore, the concentrations used in our assays represent between 5–20 fold above the published endogenous levels. To better understand the mechanism of Hsp90-mediated FN internalisation, our current data extend this study and suggest that Hsp90 is involved in processes which maintain the stability of the extracellular FN matrix for which LRP1 mediates the clearance upon destabilisation or inhibition by NOV. To the best of our knowledge, this is the first report to investigate Hsp90 inhibition on FN turnover in MEFs and to link a dual role for Hsp90 and LRP1 in the turnover of FN. LRP1 exists in different pools in the cell, some will be intracellular (during synthesis or endocytosis) whilst the mature protein will be expressed on the cell surface. LRP1 has an extracellular region which comprises its ligand binding domains. LRP1 is known to endocytose and degrade a number of extracellular proteins including thrombospondins and plasminogen activators^50,51^ and functions as a receptor for extracellular Hsp90. Upon ectopic addition of Hsp90, it is able to bind the extracellular fragment of LRP1 and exert either cytokine like roles or chaperoning functions. Therefore, it is likely that Hsp90 (added ectopically) will interact with LRP1, as reported by others^25,29,52^, followed by internalisation of this complex (with FN) during endocytosis which gives rise to the presence of LRP1 complexes intracellularly as observed in our study. It has also previously been reported that LRP1 is able to mediate FN internalisation and degradation^23^. Here we show that LRP1 is required for the NOV-mediated turnover of extracellular FN.

The commercial monoclonal LRP1 blocking antibody used in our study binds the 515 kDa alpha chain of LRP1 and prevents binding of ligands^37^. We anticipated that treatment with the blocking antibody would prevent FN endocytosis and result in an accumulation of extracellular FN matrix, however, this was not the case. Instead we found that the LRP1 blocking antibody caused a loss in FN matrix similar to that observed for NOV treatment, and which was rescued by exogenous Hsp90β. FN matrix polymerization regulates the stability of FN matrix fibrils, and inhibitors of FN polymerization have been shown to diminish the amount of FN accumulating in the extracellular matrix^53^. Our data might suggest a role for LRP1 in FN fibrillogenesis and/or stabilization of FN fibril assembly, in addition to the previously described role for LRP1 in FN catabolism^23^. Irrespective of this, these data suggest that NOV treatment somehow leads to the inhibition of LRP1.

The mechanism by which NOV inhibits LRP1 remains to be determined. We showed that Hsp90 forms a common complex with FN and LRP1 on the surface of MEF-1 and Hs578T cells, which surprisingly was not perturbed by NOV. This suggests that regulation of FN turnover by LRP1 is not due to changes in this complex. It is possible that the effect may be indirect and as a result of altered downstream signalling cascades in response to NOV. Roles for Hsp90-LRP1 cross-membrane signalling has previously been reported, whereby binding of extracellular Hsp90 to LRP1 induced the activation of downstream signalling pathways involving Akt and mTOR^26^ and NFkB^28^. Activation of Akt and NFkB have been reported to increase expression of FN^32,33^ and promote FN matrix assembly by activation of integrins^54^. We might then expect a scenario where Hsp90 inhibition disrupts the Hsp90-LRP1 induced Akt or NFkB signalling to reduce FN expression leading to loss of the FN ECM. However, this interpretation was not supported by the observations in our study as FN mRNA levels did not substantially change upon NOV treatment in MEFs (data not shown), and the levels of soluble FN were reduced by proteolysis in NOV-treated cells. In addition, it is not immediately clear how downstream signalling by Hsp90-LRP1 could be affected given that there was no change in the association of these proteins. Roles for Hsp90-LRP1 mediated activation of MMPs have also been demonstrated^31,48,55,56^. MMPs are known to degrade the ECM (including FN) and activation of these enzymes has been linked to increased cell invasiveness^57,58^. However, we did not see an increase in MMPs (data not shown) upon NOV treatments in either cell lines, which suggests that this is not the mechanism.

There is still debate around whether Hsp90 functions as a chaperone in the extracellular space or acts rather as a signalling molecule. We hypothesize that Hsp90 may have roles in both intracellular stabilization of soluble FN destined for export and extracellular stabilization of insoluble FN matrix and/or assisting in matrix assembly by regulating signalling pathways as a potential cytokine. These pathways still need to be identified and investigation into the precise mechanisms by which Hsp90 and LRP1 regulates NOV-induced FN turnover is continuing. The ECM is highly dynamic and is constantly being remodelled in both normal and diseased states. The functional relevance of the FN matrix and its turnover, in particular whether it serves to promote cell invasiveness by providing a scaffold on which to migrate, or acts as a barrier to migration, is an ongoing debate. However, it is clear that changes in the FN ECM are associated with pathology. The fact that certain Hsp90 inhibitors, which are intended for clinical use, cause deregulation of FN via a receptor that is ubiquitously expressed, means that these inhibitors may induce unintended ECM remodelling in a range of cell types which could ultimately culminate in disease. Further investigation is currently underway, but we have preliminary evidence which demonstrates an increased ability of LRP1-expressing cells to migrate at certain concentrations of NOV, suggesting putative physiological consequences associated with FN remodelling due to Hsp90 inhibition.

## Methods

### Antibodies

Mouse monoclonal anti-human Hsp90α/β (cat no.: sc-13119) and goat polyclonal anti-human Hsp90α/β (cat no: sc-1055) primary antibodies were from Santa Cruz Biotechnology (USA). Mouse anti-human fibronectin (cat no.: F0916), rabbit anti-human fibronectin (cat no.: F3648), rabbit anti-human actin (cat no.: A2103) primary antibodies and goat anti-mouse IgG-HRP (cat no.: A2304) secondary antibody were from Sigma Aldrich (Germany). Rabbit monoclonal anti-human LRP1 (ab92544), mouse monoclonal [TV.1] anti-human fibronectin (ab194395), rabbit polyclonal anti-human histone H3 (ab1791) primary antibodies and donkey anti-rabbit IgG-HRP (ab16284) secondary antibody were from Abcam (UK). Donkey anti-mouse Dylight® 488 (ab96875), donkey anti-rabbit Dylight® 555 (ab96892) and donkey anti-goat Dylight® 650 (ab96934) secondary antibodies were also from Abcam (UK). Mouse monoclonal anti-human LRP1 blocking antibody (GTX79843) was from GeneTex (USA). Alexa Fluor-488 conjugated donkey anti-mouse IgG (cat no.: A21202), Alexa Fluor-546 conjugated donkey anti-rabbit IgG (cat no.: A10040), Alexa Fluor-660 conjugated donkey anti-goat IgG (cat no.: A21082) were from Invitrogen (UK).

### Cell Culture

Hs578T (ATCC: HTB-126) breast cancer cell line and mouse embryonic fibroblasts (MEF) (i.e. LRP1 wild type MEF-1 [ATCC: CRL-2214] and LRP1 deficient PEA-13 [ATCC: CRL-2216]) were purchased from the American Type Culture Collection (ATCC). Hs578T breast cancer cells were maintained in Dulbecco’s Modified Eagle’s Medium (DMEM) supplemented with 10% [v/v] FCS, 2 mM GlutaMAX™, 100 U/ml PSA and 2 mM insulin (Novorapid, Canada). Murine embryonic fibroblast lines (MEF) were maintained in DMEM supplemented with 10% [v/v] FCS, 2 mM GlutaMAX™ and 100 U/ml PSA. All cell lines were maintained at 37 °C in a humidified atmosphere with 9% CO_2_.

### SDS-PAGE and immunoblotting

Proteins were separated by discontinuous SDS-PAGE according to the modifications of the method described by Laemmli^59^. Resolved proteins were transferred onto nitrocellulose membrane in western transfer buffer (25 mM Tris-Cl, 192 mM glycine, 20% [v/v] methanol) for 50 minutes at 0.4 A. Membranes were blocked for at least 1 hour at room temperature in 5% BLOTTO (5% [w/v] non-fat milk powder in Tris buffered saline [TBS: 50 mM Tris, 150 mM NaCl pH 7.5]). Membranes were incubated with primary antibody in 1% [w/v] BLOTTO at the recommended dilution overnight at 4 °C. Membranes were subsequently washed several times in TBST (TBS with 0.1% [v/v] Tween-20). Species matched secondary antibody conjugated to HRP was incubated with the membrane in fresh 1% [w/v] BLOTTO for 45 minutes at room temperature shaking after which the membranes were washed again in TBST at least four times. Proteins were detected using the ECL Advanced western blotting detection kit and visualised on the Chemidoc™ XRS system (BioRad). The full length Western blots for respective figures can be found in the Supplementary File.

### Confocal Microscopy

Cells were seeded (3 × 103 cells/well) into a 15-well ibidi plate and incubated overnight to allow cells to adhere. Cells were washed in phosphate buffered saline (PBS: pH 7.4; 137 mM NaCl, 27 mM KCl, 4.3 mM Na2HPO4, 4 mM KH2PO4), flash-treated with ice-cold ethanol and air dried. Cells were blocked with 1% [w/v] BSA/TBS and incubated with primary antibodies in 1% [w/v] BSA/TBS overnight at 4 °C. Cells were washed twice with 0.1% [w/v] BSA/TBS followed by 1 hour incubation with species specific fluorescently tagged secondary antibodies. Antibodies used are specified in figure legends. Nuclei were stained with Hoechst-33342 dye (Invitrogen) (1 μg/ml in distilled water). Images were captured using the Zeiss LSM780 Meta laser scanning confocal microscope and analyzed using Zen Blue Software.

### Biochemical fractionation of insoluble and soluble fibronectin using a deoxycholate (DOC) assay

This assay was adapted from that published by Brenner and colleagues^44^. MEF-1 and PEA-13 cells were seeded in a 6-well plate (6 × 105 cells/well) and allowed to adhere overnight. Cells were treated with increasing concentrations of NOV for 16 hours and scraped into DOC buffer (2% [w/v] deoxycholate, 20 mM Tris-HCl, pH 8.8, 2 mM phenylmethanesulfonylflouride [PMSF], 2 mM EDTA and 0.05% [v/v] protease inhibitor cocktail). To separate soluble and insoluble fractions, the samples were vortexed for 2 minutes and centrifuged at 13000 rpm in a microfuge for 20 minutes at 4 °C. The supernatant containing soluble fibronectin was removed and the cell pellet containing insoluble fibronectin was resuspended in SDS sample buffer (1% [w/v] SDS, 25 mM Tris-HCl, pH 8.0, 2 mM PMSF, 2 mM EDTA and 0.05% [v/v] protease inhibitor cocktail).

### Cell surface biotinylation and streptavidin affinity purification

Adherent MEF-1 and PEA-13 cells were treated for 16 hours with or without 200 μM NOV at 37 °C. Cell surface proteins were biotinylated by incubation with 1 mg/ml EZ-Link™ Sulfo-NHS-SS-Biotin in PBS (pH 8) for 1 hour at 4 °C and quenched in 1 M Tris-Cl (pH 7.5). Cells were washed twice in PBS to remove unbound NHS-biotin and lysed in radio-immunoprecipitation assay (RIPA) buffer (50 mM Tris-Cl, pH 7.4, 150 mM NaCl, 1 mM EDTA, 1 mM Na3VO4, 1% [v/v] Nonidet P-40 [NP40], 1 mM sodium deoxycholate, 1 mM PMSF, 0.05% [v/v] protease inhibitor cocktail) with gentle scraping. A second biotinylated flask (negative control) was lifted with trypsin/EDTA (to cleave surface protein interactions) followed by centrifugation at 2000 rpm for 2 minutes in a microfuge. Pelleted cells were resuspended in RIPA buffer. Biotinylated scraped or trypsinised cells were lysed for 30 minutes at 4 °C with gentle agitation. Lysates were cleared by centrifugation in a microfuge at 13000 rpm for 5 minutes at 4 °C and incubated with streptavidin conjugated agarose beads (Thermo Scientific, USA) for 1 hour at 4 °C. After centrifugation, the supernatant was discarded and purified proteins were released from the beads by boiling in 5x SDS sample buffer. Samples were resolved by SDS-PAGE and analysed by immunoblotting as described previously.

### DTSSP cell surface crosslinking and immunoprecipitation of LRP1 containing complexes

Adherent cells were left untreated or treated with NOV as indicated in figure legends. Cells were washed twice in PBS and incubated with 3,3′-Dithiobis(sulfosuccinimidylpropionate) (DTSSP, 3 mg/ml) at 4 °C for 2 hours to allow for crosslinking of protein interactions followed by quenching with 1 M Tris-Cl (pH 7.5) at 4 °C for 15 minutes. Cells were lysed in ice cold non-denaturing buffer (20 mM Tris-Cl, pH 8, 137 mM NaCl, 1% [v/v] NP40, 2 mM EDTA, 0.05% [v/v] protease inhibitor cocktail) with gentle scraping. MagReSyn™ Protein A was bound to rabbit anti-human LRP1 primary antibody or an isotype control antibody according to manufacturer’s instructions (ReSyn Biosciences, South Africa) and subsequently incubated with cleared lysates overnight at 4 °C. Beads in suspension were collected using a magnet and washed three times in wash buffer (50 mM Tris, pH 7, 150 mM NaCl, 1% [v/v] Tween 20) followed by a final wash in distilled water. LRP1 complexes were eluted from the beads by boiling in 5x SDS sample buffer containing β-mercaptoethanol to cleave DTSSP bound proteins from the LRP1 complex. Immunoprecipitated proteins were analysed by SDS-PAGE and immunoblotting as described.

### Statistical analysis

Experiments were performed in triplicate unless otherwise stated and statistical analyses were conducted using either one-way or two-way ANOVA with Bonferroni post-test or unpaired two-tailed Students t-tests in GraphPad Prism Version 4 software.

### Data availability

The datasets generated during and/or analysed during the current study are available from the corresponding author on reasonable request.

## Electronic supplementary material

Supplementary figures
