# Author Correction: LRP1 is required for novobiocin-mediated fibronectin turnover

**DOI:** 10.1038/s41598-020-65538-4

**Published:** 2020-05-15

**Authors:** Natasha Marie-Eraine Boel, Morgan Campbell Hunter, Adrienne Lesley Edkins

**Affiliations:** Biomedical Biotechnology Research Unit, Department of Biochemistry and Microbiology, Rhodes University, Grahamstown, 6140 South Africa

Correction to: *Scientific Reports* 10.1038/s41598-018-29531-2, published online 30 July 2018

This Article contains an error. The BSA/UNT panels in Figure 1 were inadvertently duplicated from the BSA/NOV condition, which also affected the MGV quantification for these conditions. The correct Figure 1 appears below, with the MGV re-calculated.

**Figure 1:**
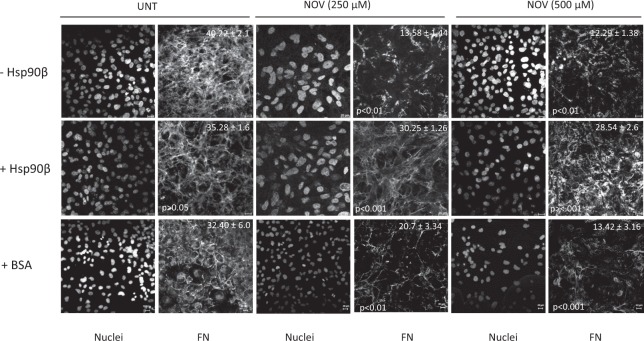
.

