# Supplementary material for: LRP1 is required for novobiocin-mediated fibronectin turnover
Source: Sci Rep. 2018 Jul 30;8:11438. doi: 10.1038/s41598-018-29531-2 (PMC6065439; doi:10.1038/s41598-018-29531-2)
Supplement: Supplementary file 1 — Supplementary figures [file 41598_2018_29531_MOESM1_ESM.docx]

**LRP1 is required for novobiocin-mediated fibronectin turnover**

Natasha Marie-Eraine Boel, Morgan Campbell Hunter and Adrienne Lesley Edkins*

Biomedical Biotechnology Research Unit, Department of Biochemistry and Microbiology, Rhodes University, Grahamstown 6140, South Africa

*Corresponding author contact details:

[a.edkins@ru.ac.za](mailto:a.edkins@ru.ac.za)

**
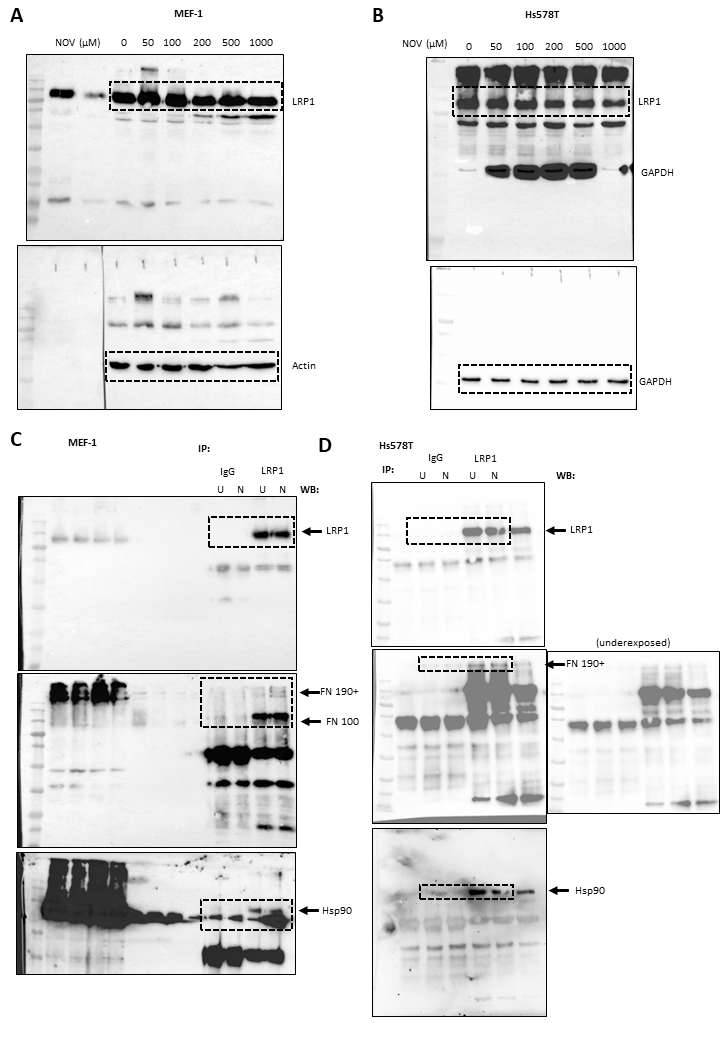
**

**Supplementary Figure 1: Full length western blots for Figure 5**

Dotted boxed areas illustrate protein bands of interest displayed in Figures.

**
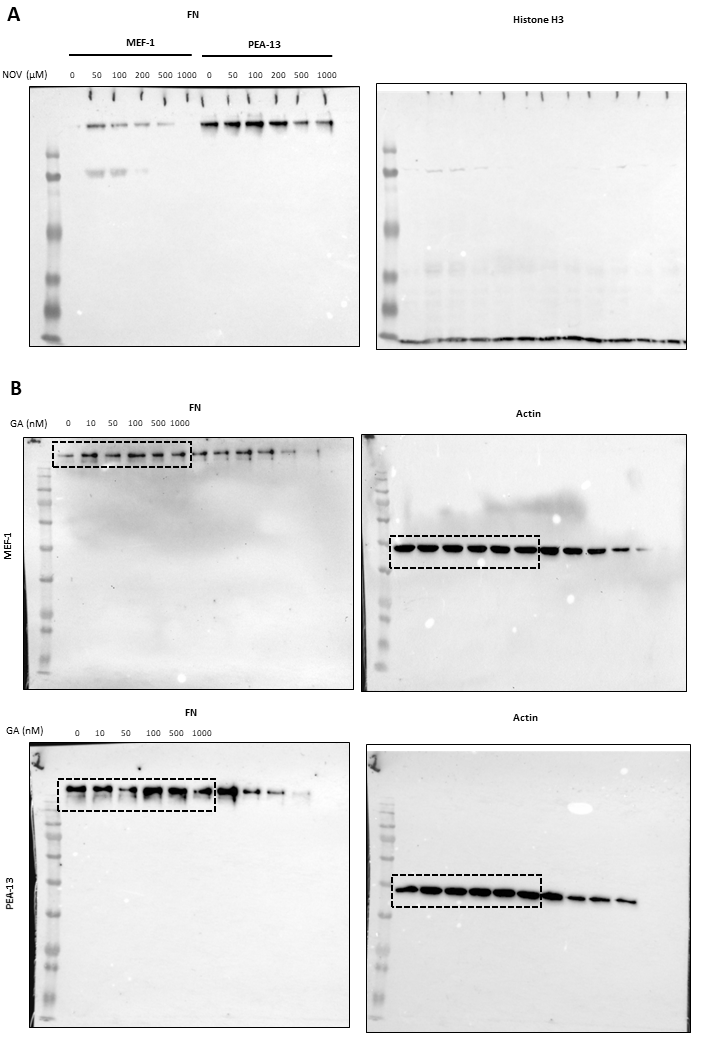
**

**Supplementary Figure 2: Full length western blots for Figure 7**

Dotted boxed areas illustrate protein bands of interest displayed in Figures.

**
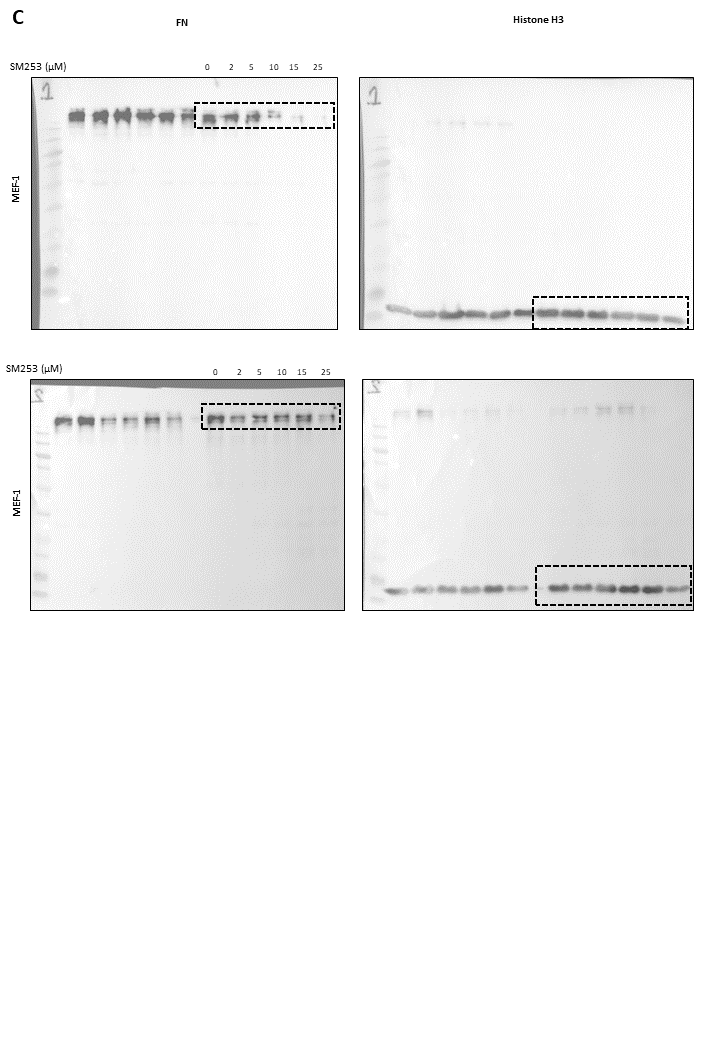
Supplementary Figure 3: Full length western blots for Figure 7**

Dotted boxed areas illustrate protein bands of interest displayed in Figures.


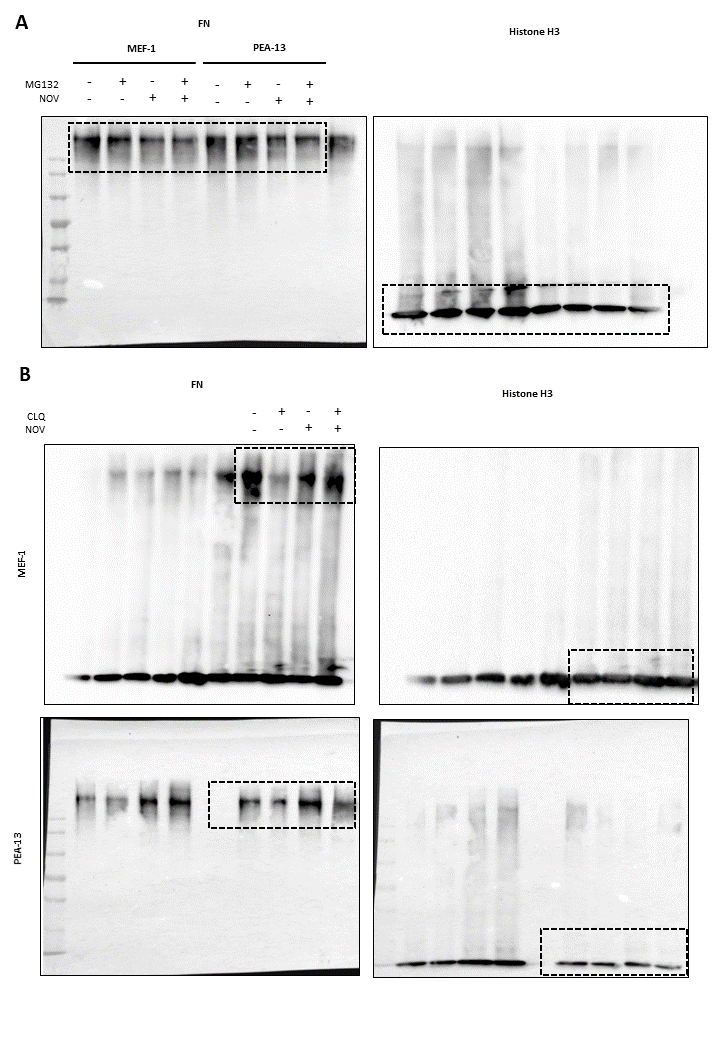


**Supplementary Figure 4: Full length western blots for Figure 8**

Dotted boxed areas illustrate protein bands of interest displayed in Figures


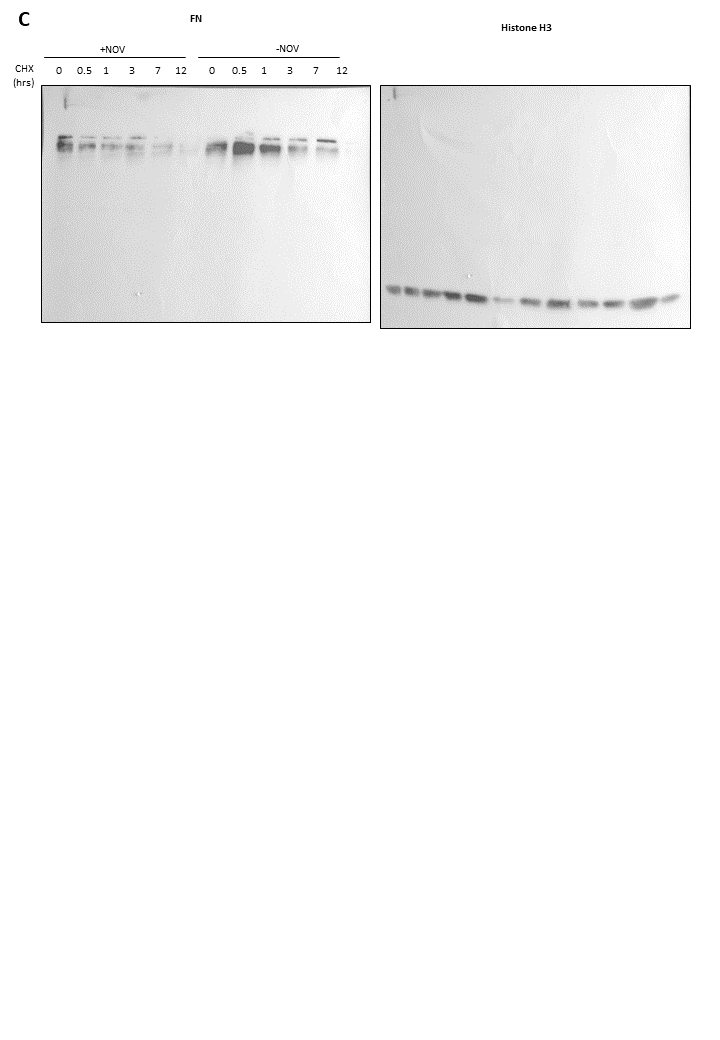
**Supplementary Figure 5: Full length western blots for Figure 8**

Dotted boxed areas illustrate protein bands of interest displayed in Figures


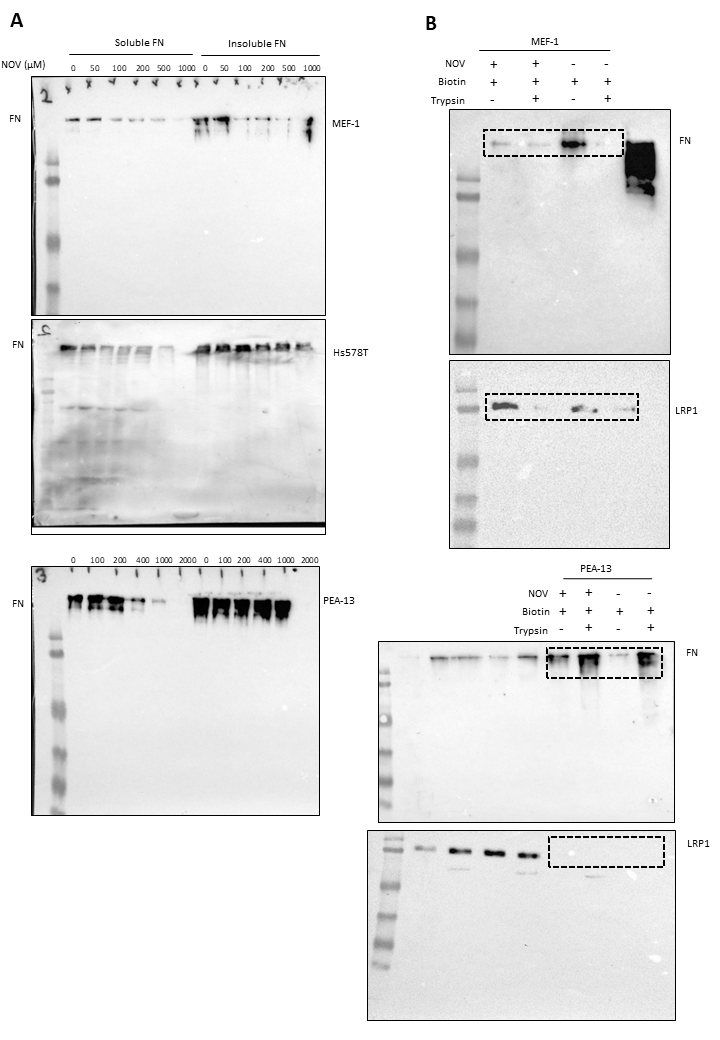


**Supplementary Figure 6: Full length western blots for Figure 9**

Dotted boxed areas illustrate protein bands of interest displayed in Figures
